# Essential palatal myoclonus following dental surgery: a case report

**DOI:** 10.1186/1752-1947-7-241

**Published:** 2013-10-14

**Authors:** Jeff H Lam, Mairi E Fullarton, Alex MD Bennett

**Affiliations:** 1 College of Medicine, University of Edinburgh, 47 Little France Crescent, Edinburgh EH16 4TJ, UK; 2 Department of Otolaryngology, Lauriston Building, Lauriston Place, Edinburgh EH3 9HA, UK

**Keywords:** Botulinum injection, Dental surgery, Essential palatal myoclonus, Muscle spasm, Soft palate, Tinnitus

## Abstract

**Introduction:**

Various presentations of essential palatal myoclonus, a condition characterized by clicking noises and palatal muscle spasm, have been reported in the literature. We are reporting the first case of essential palatal myoclonus following dental treatment.

**Case presentation:**

A 31-year-old Caucasian man presented to our Ear, Nose and Throat department complaining of objective clicking tinnitus occurring immediately after he had undergone root canal treatment on his right lower third molar 3 months ago. Magnetic resonance imaging of his head revealed no abnormalities in the cerebrum, cerebellum or brainstem making the diagnosis essential palatal myoclonus. He returned a week later, and 20 units of botulinum toxin A (Allergan) were injected into his left tensor veli palatine muscle. He reported an immediate improvement; however, symptoms recurred 6 months later.

**Conclusions:**

Dental treatment can be a trigger of essential palatal myoclonus. Botulinum toxin injections are an effective treatment for short-term relief of symptoms.

## Introduction

Palatal myoclonus (PM; or palatal tremor) is a rare condition affecting the muscles of the soft palate. It typically presents with clicking noises and muscle spasms felt at the back of the throat. PM is divided into two subtypes: essential (EPM) and symptomatic (SPM). EPM describes an objective clicking secondary to rhythmic movements of the tensor veli palatini (TVP), occurring intermittently throughout the day but not during sleep [1]. Previous literature demonstrates that EPM typically presents in late childhood [2,3], and occurs in the absence of any cerebellar or brainstem lesion.

**Figure 1:**
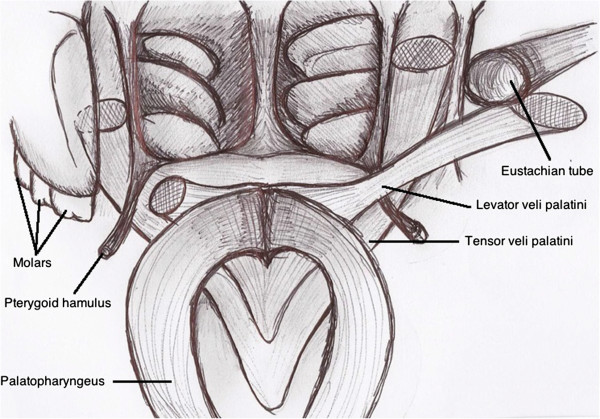
Anatomy of the soft palate.

SPM involves the levator veli palatini muscles (Figure 1), and is usually caused by a lesion in the Guillain–Mollaret triangle, which comprises the dentate, red and inferior olivary nuclei [4]. In contrast to EPM, SPM does cause symptoms during sleep.

We report the first case of EPM following dental treatment.

## Case presentation

A 31-year-old Caucasian man presented to our Ear, Nose and Throat department complaining of bilateral objective clicking tinnitus occurring immediately after he had undergone root canal treatment on his right lower third molar 3 months ago. The dental surgery was uneventful but the patient felt his mouth was over-extended during the procedure. The tinnitus was constant and he could not exert any control over his symptoms. He reported his symptoms were bilateral but alternating in severity between the right and left sides. As the patient lived alone, it was not known whether his symptoms occurred during sleep. He denied any symptoms of the pharynx, larynx or esophagus or any other auditory symptoms. He appeared anxious but there was no significant past medical history. He was not taking any regular medications.

Examination of the patient revealed objective clicking predominantly on the right side. On inspection of his oral cavity, muscle spasms of his soft palate were observed. He was subsequently sent for magnetic resonance imaging of his head, which revealed no abnormalities in the cerebrum, cerebellum or brainstem making the diagnosis EPM. He returned a week later, and 20 units of botulinum toxin A (Allergan) were injected into his left tensor veli palatine muscle. He reported an immediate improvement with no side effects; however, symptoms had recurred at follow-up 6 months later. He was subsequently referred for consideration of radio-frequency ablation with the aim of achieving long-term symptom relief.

## Discussion

As previously mentioned, the primary muscle affected in EPM is the TVP. The left and right TVP share a common aponeurosis which extends between the pterygoid hamuli. This anatomical connection provides an explanation for the typically bilateral symptoms of EPM [5].

Psychogenic PM is a subtype of EPM which has a similar presentation. Physical triggers for the condition such as endoscopy have been reported in this condition [6]. However, the primary factor in distinguishing psychogenic PM is the ability of the patient to exert voluntary control over his or her symptoms [7] and our patient was unable to demonstrate this, making a psychogenic cause for his symptoms unlikely.

The exact precipitant of PM in this case is unclear. Wisdom tooth surgery is occasionally complicated by damage to the branches of the mandibular nerve, most commonly the inferior alveolar and lingual nerves, but not the medial pterygoid nerve which supplies the TVP. However, this case raises the possibility of medial pterygoid nerve damage following dental surgery as a cause of EPM.

Botulinum toxin is the only pharmacological therapy which has consistently been proven to be effective in the treatment of PM [8]. Up to 30 units are injected unilaterally into the soft palate at the site of insertion of the TVP and levator [9]. Side effects of treatment are usually brief and include voice change and nasopharyngeal regurgitation [10]. More severe side effects are rare and include Eustachian tube dysfunction and velopharyngeal inadequacy [8].

Systemic treatments that have been evaluated in the management of PM include phenytoin, barbiturates, benzodiazepines, carbamazepine, sodium valproate and anticholinergics, however, many of these drugs are associated with troublesome sedative and metabolic side effects [11]. A previous case report has shown that radio-frequency ablation may be effective in abolishing abnormal movements of the soft palate [12].

## Conclusions

Dental treatment can be a trigger of EPM. Botulinum toxin injection is an effective treatment for short-term relief of symptoms.

## Consent

Written informed consent was obtained from the patient for publication of this case report and any accompanying images. A copy of the written consent is available for review by the Editor-in-Chief of this journal.

## Abbreviations

EPM: Essential palatal myoclonus; PM: Palatal myoclonus; SPM: Symptomatic palatal myoclonus; TVP: Tensor veli palatini.

## Competing interests

The authors declare that they have no competing interests.

## Authors’ contributions

AB was the consultant responsible for diagnosing and treating the patient in this case report. AB provided the information to JL and MF, who wrote the paper under AB’s supervision. All authors have read and approved the final version of this manuscript.

## References

[B1] Pearce JM Palatal Myoclonus (syn. Palatal Tremor) Eur Neurol 2008 60 6 312 315 10.1159/000159929 18832845

[B2] Camistol-Plana J Majumdar A Fernández-Alvarez E Palatal tremor in childhood: clinical and therapeutic considerations Dev Med Child Neural 2006 48 982 984 10.1017/S0012162206002155 17109787

[B3] Schwartz RH Bahadori RS Myseros JS Loud clicking sounds associated with rapid soft palate muscle contractions Pediatr Emer Care 2012 28 158 159 10.1097/PEC.0b013e3182443000 22307183

[B4] Guillain G Mollaret P Bertrand I Sur la lesion responsible du syndrome myoclonique du tronc cerebral Rev Neurol (Paris) 1993 3 666 674

[B5] Deuschl G Toro C Hallett M Symptomatic and essential palatal tremor. 2. Differences of palatal movements Mov Disord 1994 9 6 676 678 10.1002/mds.870090615 7845410

[B6] Stamelou M Saifee TA Edwards MJ Bhatia KP Psychogenic palatal tremor may be underrecognized: reappraisal of a large series of cases Mov Disord 2012 27 9 1164 1168 10.1002/mds.24948 22434706 PMC4235251

[B7] Ross S Jankovic J Palatal myoclonus: an unusual presentation Mov Disord 2005 20 9 1200 1203 10.1002/mds.20516 15929094

[B8] Bryce GE Morrison MD Botulinum toxin treatment of essential palatal myoclonus tinnitus J Otolaryngol 1998 27 4 213 216 9711516

[B9] Karuse E Heinen F Gurkov R Difference in outcome of botulinum toxin treatment of essential palatal tremor in children and adults Am J Otolaryngol 2010 31 2 91 95 10.1016/j.amjoto.2008.11.007 20015723

[B10] Penney SE Bruce IA Saeed SR Botulinum toxin is effective and safe for palatal tremor: a report of five cases and a review of the literature J Neurol 2006 253 7 857 860 10.1007/s00415-006-0039-9 16845571

[B11] Chitkara A Cultrara A Blitzer A Palatal myoclonus: treatment with botulinum toxin Operative Tech in Otolaryngol – Head Neck Surg 2004 15 2 114 117 10.1016/j.otot.2004.01.012

[B12] Aydin O Iseri M Ozturk M Radiofrequency ablation in the treatment of idiopathic bilateral palatal myoclonus: a new indication Ann Otol Rhinol Laryngol 2006 115 11 824 826 17165664 10.1177/000348940611501105

